# Feature-Based Retinal Image Registration Using D-Saddle Feature

**DOI:** 10.1155/2017/1489524

**Published:** 2017-10-24

**Authors:** Roziana Ramli, Mohd Yamani Idna Idris, Khairunnisa Hasikin, Noor Khairiah A. Karim, Ainuddin Wahid Abdul Wahab, Ismail Ahmedy, Fatimah Ahmedy, Nahrizul Adib Kadri, Hamzah Arof

**Affiliations:** ^1^Department of Computer System & Technology, Faculty of Computer Science & Information Technology, University of Malaya, Kuala Lumpur, Malaysia; ^2^Department of Biomedical Engineering, Faculty of Engineering, University of Malaya, Kuala Lumpur, Malaysia; ^3^Regenerative Medicine Cluster and Imaging Unit, Advanced Medical & Dental Institute, Universiti Sains Malaysia, Pulau Pinang, Malaysia; ^4^Faculty of Medicine & Health Sciences, Universiti Malaysia Sabah, Sabah, Malaysia; ^5^Department of Electrical Engineering, Faculty of Engineering, University of Malaya, Kuala Lumpur, Malaysia

## Abstract

Retinal image registration is important to assist diagnosis and monitor retinal diseases, such as diabetic retinopathy and glaucoma. However, registering retinal images for various registration applications requires the detection and distribution of feature points on the low-quality region that consists of vessels of varying contrast and sizes. A recent feature detector known as Saddle detects feature points on vessels that are poorly distributed and densely positioned on strong contrast vessels. Therefore, we propose a multiresolution difference of Gaussian pyramid with Saddle detector (D-Saddle) to detect feature points on the low-quality region that consists of vessels with varying contrast and sizes. D-Saddle is tested on Fundus Image Registration (FIRE) Dataset that consists of 134 retinal image pairs. Experimental results show that D-Saddle successfully registered 43% of retinal image pairs with average registration accuracy of 2.329 pixels while a lower success rate is observed in other four state-of-the-art retinal image registration methods GDB-ICP (28%), Harris-PIIFD (4%), H-M (16%), and Saddle (16%). Furthermore, the registration accuracy of D-Saddle has the weakest correlation (Spearman) with the intensity uniformity metric among all methods. Finally, the paired *t*-test shows that D-Saddle significantly improved the overall registration accuracy of the original Saddle.

## 1. Introduction

Retinal image registration includes the process of aligning target (moving) image to the orientation of reference (fixed) image. The alignment is performed according to the transformation estimated based on the corresponding information between retinal images. The retinal image registration is typically employed in super-resolution, image mosaicking, and longitudinal study applications. Super-resolution combines information from multiple images with large overlapping area to increase density of spatial sampling and improve pathological information. Furthermore, super-resolution can resolve the blurring edges on retinal vessels due to eye movements during image acquisition. In image mosaicking, retinal images with small overlapping area are aligned to generate a wider view of the retina as an ophthalmic camera has a limited field of view between 30° and 50° at a time. Through image mosaicking, ophthalmologist can display the retina in one big picture and this is beneficial to illustrate the full extent of the retinal disease in adult or neonatal for optimum diagnosis [1, 2]. Furthermore, mosaicking application has been explored in eye laser treatment for diabetic retinopathy [3]. In longitudinal study application, retinal images captured from different time are utilized in the registration. The longitudinal study application is important to monitor the progression of eye diseases such as glaucoma and age-related macular degeneration that usually undergoes a long degeneration process [4].

Prior works on retinal image registration can be classified as area-based and feature-based approaches. The area-based approach estimates the transformation by comparing intensity patterns between fixed and moving images via similarity metrics such as mutual information [5], cross correlation, sum of absolute values of differences, and phase correlation [6]. Through optimization process, the similarity metric considers all intensity patterns in the images and iteratively refines the initial transformation parameters until the optimum registration is obtained. However, optimizing the similarity metric using all intensity patterns in the images is computationally expensive. Furthermore, intensity patterns in nonoverlapping area particularly in an image pair with small overlap area may mislead the similarity metric and results in inaccurate registration. Additionally, area-based approach is sensitive to significant background and anatomical changes over time [5, 7]. For example, progression in a glaucoma patient will alter the topographic of optic disc over time. For example, progression in a glaucoma patient will alter the topographic of optic disc over time, thus significantly changes the intensity pattern between the image pair and leads to inaccurate registration.

Feature-based approach searches for the transformation according to the correspondence features across images. There are four main components in feature-based approach, namely, detecting features, assigning descriptors, matching the corresponding features, and searching the transformation between images. The feature-based approach is more robust to the changes of intensity, scale, and rotation than the area-based approach but requires stable and repeatable features between images [8]. The widely used feature in feature-based approach is vessel bifurcations [9–13] that can be detected through branch point analysis of segmented vascular tree. Then, vessel bifurcations are characterized with intensity orientations or surrounding vessel branch information. However, efficient detection of vessel bifurcations requires a reliable vascular tree segmentation technique and can be a challenging task in low-quality and unhealthy retinal image. Furthermore, the sparse and uneven distribution of vessel bifurcations can lead to inaccurate registration of image pair with small overlap area.

Alternatively, a feature-based approach using local feature is independent of vascular segmentation which is stable and distinctive. The local feature finds the extrema or the changes of intensity level in local patches to detect interest points. Then, these points are matched across images. To reduce false matches, algorithms such as random sample consensus (RANSAC) [14] and M-estimator sample consensus (MSAC) [15] are utilized to obtain inliers. Finally, the inliers are used to estimate the transformation between images. Among the local feature methods considered in existing retinal image registration are Harris corner [16], speeded-up robust features (SURF) [17, 18], and scale invariant feature transform (SIFT) [19].

Chen et al. detected Harris corner points and assigned partial intensity invariant feature (Harris-PIIFD) descriptor to each point relative to the main orientation (0, *π*) of local gradient [20]. Harris-PIIFD was tested on low-quality multimodal retinal images. This method can successfully register retinal images when the overlapping area is above 30% and low-quality retinal images in which the vasculature is hard to extract. However, Harris corner has low repeatability rate in the presence of anatomical changes between the retinal image pair. Lack of repeatable feature points throughout the images can lead to inaccurate or failed registration.

The issue of low repeatability rate in Harris-PIIFD is addressed in SURF–PIIFD–RPM by considering SURF and robust point matching to reject a large number of outliers [21]. However, their success registration rate decreases to 50% when the overlapping area is below 50%. SURF in retinal image registration is further explored in [22] by directing the detection of SURF points on vessels as vessels are reliable over time even in unhealthy retinal image. Conversely, Hernandez-Matas et al. extracted the SURF points from all over retinal image [23] and their work is further improved in [24] by utilizing SIFT points. In retinal image, SURF is reliable and fast to compute but SIFT has a higher localization accuracy than SURF.

Generalized dual-bootstrap iterative closest point (GDB-ICP) [25] algorithm utilized SIFT points to generate initial transformation and requires a minimum of one correct initial match to register the retinal image pair. GDB-ICP is very effective and widely used to register low-quality retinal images. However, GDB-ICP is highly susceptible in the presence of anatomical changes and very low-quality image. Furthermore, the distribution of feature points in GDB-ICP is severely affected by noise. Despite the high localization accuracy, SIFT suffers from the issues of quantity and distribution. These issues are addressed in UR-SIFT-PIIFD by detecting uniform robust scale invariant feature transform (UR-SIFT) points and compute the PIIFD descriptor to register noisy and low-quality retinal image [26]. The selection criterion in their work is further extended in [27] to detect points which lie on the retinal vessels based on the difference of Gaussian (DoG) values and Frangi's vesselness measure (FVM) for registration of high-resolution and low-contrast retinal images.

In prior feature-based approaches, their performances are limited in the presence of low-quality images such as illumination artifact near the frame boundary, nonuniform intensity, and dark spot artifact obscuring underlying tissues caused by poor dilated pupils. Low-quality retinal images are inevitable in unhealthy retinas caused by various diseases. Furthermore, capturing high-quality retinal images require a combination of skills and experiences of the operator to adjust the camera settings as well as cooperation from the patient itself. This will restrict their practical utilization for super-resolution, image mosaicking, and longitudinal study applications. These applications are crucial in diagnosis and monitoring retinal diseases, such as diabetic retinopathy, glaucoma, and age-related macular degeneration [4]. Therefore, feature points should be detected on the low-quality region that consists of high and low contrast vessels of varying sizes to ensure a uniform distribution of feature points on the retinal image. Highly distributed feature points on the retinal image are important to estimate an optimal transformation between the retinal image pair [22].

**Figure 1:**
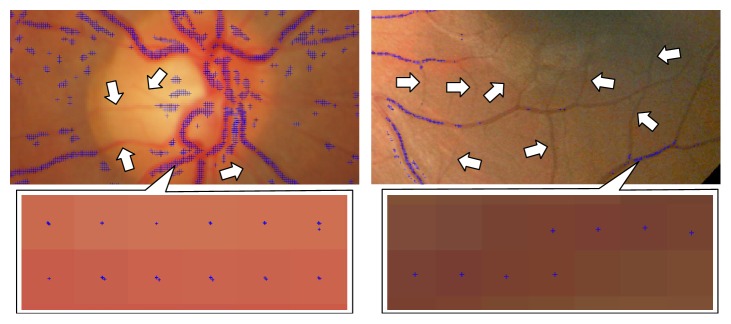
Example of Saddle points detected on fundus retinal images. Low-contrast vessels pointed by white arrows are undetected by Saddle. Small sections of the vessels are zoomed to show Saddle points with subpixel precision.

A recent local feature detector in image processing field known as Saddle detects local structures that have concave and convex profiles of a 3D intensity surface within a defined neighborhood [28]. In fundus retinal image, Saddle detects feature points on vessels but these points are poorly distributed and mainly located on strong contrast vessels as shown in Figure 1. This issue is contributed by the factor that Saddle lacks appropriate filter to emphasize the vessel structure in low-quality region of the retinal image. Another issue in Saddle is the feature points detected on vessels are densely positioned to each other and may be characterized by a similar vector descriptor. This can lead to false matches and inaccurate registration. Furthermore, Saddle utilizes the same image dimensions throughout the levels in scale-space pyramid that will increase the running time and memory usage during the detection process.

Therefore, in this study, we propose a local feature detector for retinal image registration (D-Saddle) by incorporating multiresolution DoG pyramid with Saddle detector [28] to enable the detection of Saddle points on low-quality region that consists of high and low contrast vessels of varying sizes. This will increase the distribution of feature points throughout the retinal image pair thus allowing D-Saddle to be more efficient in registering low-quality retinal image for super-resolution, image mosaicking, and longitudinal study applications.

The remainder of this paper is organized as follows. Methodology describes the details of the proposed work which includes detection, descriptor, matching, and estimating transformation. In Results and Discussion, we assess the performance of the proposed work and report the findings. Finally, the study is concluded and the future works are highlighted in Conclusion.

## 2. Methodology

The original implementation of Saddle localizes the interest points on grayscale images from scale-space pyramid. The scale-space pyramid in Saddle contains 6 levels of blur images with the same dimensions. The images are blurred with the scaling factor of 1.3 which reduces noise and detail in retinal images. The purpose of blurring the images in an increasing manner is to detect points on the structure of various sizes. In every level of the pyramid, the candidate points are extracted and tested for Saddle patterns. The pattern tests are applied on inner and outer rings surrounding the candidate points. Each candidate point that passes the pattern tests will be assigned with a response strength and followed by nonmaxima suppression step. However, nonmaxima suppression step is only applied to the candidate points located on the first level as the edge in the remaining levels is relatively coarse. Finally, subpixel precision for each point is computed over a 3-by-3 neighborhood.

### 2.1. Proposed Algorithm

**Figure 2:**
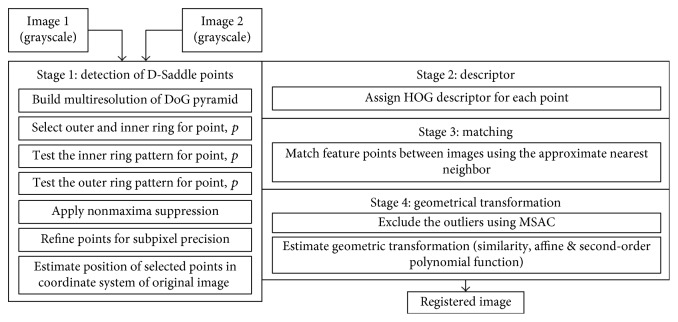
Framework of the proposed D-Saddle for fundus retinal image registration.

Figure 2 shows the registration framework for the proposed D-Saddle that comprises of four stages which are described as follows. Stage 1 involves the process of detecting feature points on a retinal image pair which highlights the key contribution of this study. Stage 2 assigns a descriptor to each feature point detected in stage 1. Stage 3 finds the match between feature points in the retinal image pair. Stage 4 excludes outliers from the matches and estimates the transformation between the image pair to perform image registration.

#### 2.1.1. Stage 1: Detection of D-Saddle Points

This stage highlights the key contribution of this study. The proposed D-Saddle detector involves seven important steps describe as follows.

**Step 1 .**

Build multiresolution DoG pyramid: DoG function is an approximation of Laplacian of Gaussian (LoG) and second-order derivative of edge detection. It can be computed by subtracting two different versions of blurred images defined in (1). The blurred images are obtained through the convolution of grayscale image with a Gaussian filter of *σ* width expressed in (2).

(1) $$\begin{array}{c} D\left(x,y,\sigma \right)=G\left(x,y,k\sigma \right)*I\left(x,y\right)-G\left(x,y,\sigma \right)*I\left(x,y\right), \end{array}$$

(2) $$\begin{array}{c} G\left(x,y,\sigma \right)=\frac{1}{2\pi \sigma^{2}}e^{-\left(x^{2}+y^{2}\right)/2\sigma^{2}}, \end{array}$$

where *D*(*x*, *y*, *σ*) is the DoG image, *G*(*x*, *y*, *σ*) is the Gaussian filter of *σ* width, *I*(*x*, *y*) is the input image, *k* is the ratio of two Gaussian filters, and ∗ is the convolution operator.

The main reason for incorporating the DoG function is because of its filtering property that acts as a bandpass filter by excluding low and high frequency noises to increase the visibility of the vessels in retinal image. This will improve the detection of feature points on the vessels at low-quality region and consequently the registration accuracy of the retinal image. We construct the DoG image from the subtraction between two versions of Gaussian-filtered images with a width of *σ*_0_ = 1.0 and *kσ*_0_. We choose the ratio of two Gaussian filters *k* = 1.6 that produces a good approximation of Laplacian [29, 30].

**Figure 3:**
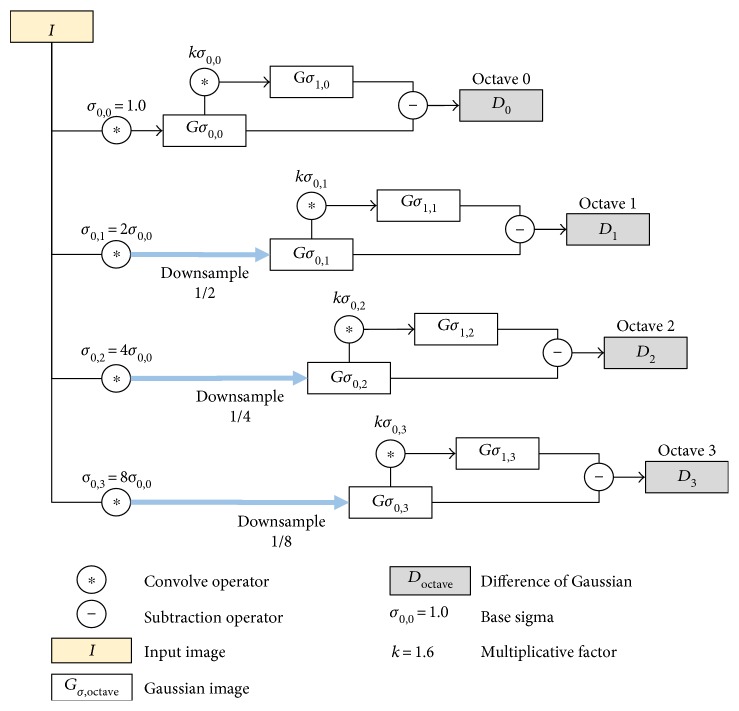
Multiresolution DoG pyramid for D-Saddle. The size of the image in subsequent octave is downsampled by half to detect feature points on vessels of varying sizes.

However, detecting feature points in the retinal image can be further challenged due to different sizes of vessels. Therefore, we utilize the multiresolution DoG pyramid to enable the detection of feature points on the different sizes of vessels. The process of building the multiresolution DoG pyramid is shown in Figure 3.

The concept of the multiresolution pyramid is to downsample the image by half the size of the previous octave creating a set of multiresolution images where the octave represents the level of the image in the pyramid. The number of the octave is set to 4 as further downsampling the image can result in a very small image that may not contain any interest points. The process of subtracting two versions of blurred images as defined in (1) is repeated in each octave to build the multiresolution DoG pyramid.

**Figure 4:**
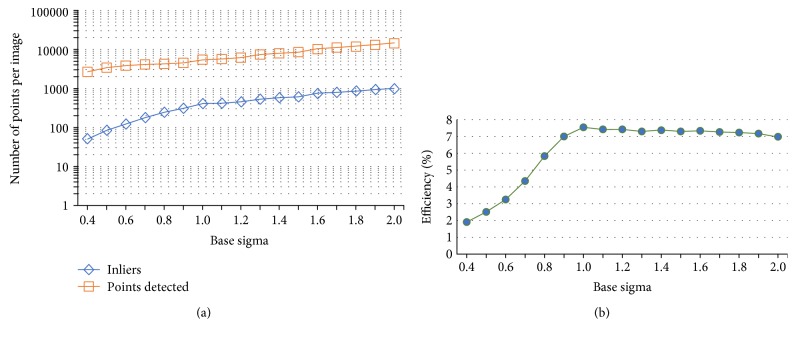
(a) The top orange line shows the average of total feature points detected per image. The lower blue line shows the average of total inliers for every base sigma *σ*_0_ tested. (b) The green line shows the efficiency given by the ratio between total inliers and total feature points detected.

**Figure 5:**
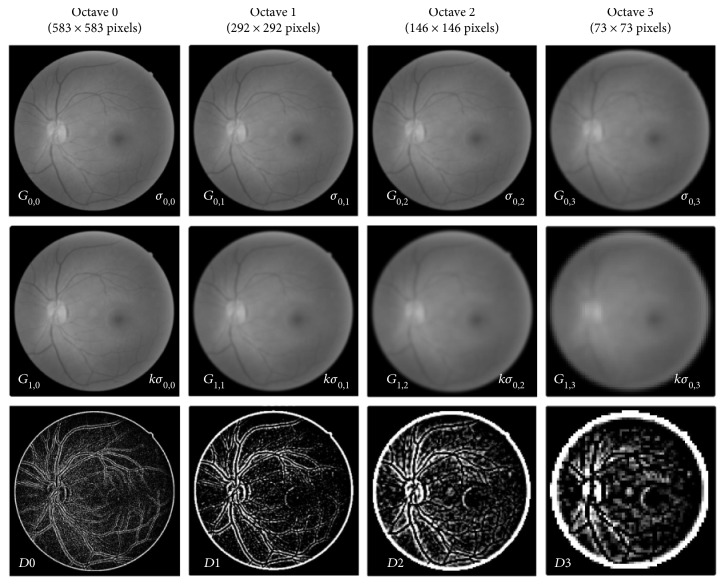
Example of retinal image in multiresolution DoG pyramid. The third row shows the absolute DoG images where black has a value of zero and white as one.

To choose the base sigma *σ*_0_ for the multiresolution DoG pyramid, we tested *σ*_0_ values between 0.4 and 2.0 with an increasing step of 0.1 on 15 retinal image pairs. From this test, the total of feature points detected and inliers per image increases with the increment of base sigma *σ*_0_ value as shown in Figure 4(). However, efficiency given by the ratio between total of inliers and feature points detected is gradually decreased when *σ*_0_ value is larger than 1.0. This shows that a larger *σ*_0_ value (>1.0) will cause more unstable feature points being detected. In image registration, a higher number of inliers can ensure a more accurate registration. However, choosing a larger *σ*_0_ value can be less efficient and computationally expensive. Therefore, by considering the trade-off between efficiency and total inliers, the base sigma *σ*_0_ = 1.0 for the multiresolution DoG pyramid is selected in this study. An example of the multiresolution DoG pyramid in fundus retinal image is depicted in Figure 5().

**Step 2 .**

**Figure 6:**
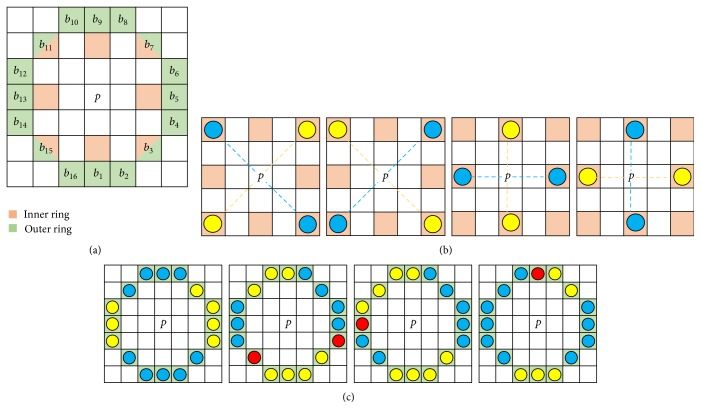
(a) Pixels' position of inner and outer rings for candidate point, *p* [28]. (b) Yellow dots represent pixels with the intensity slightly lower than the intensity in blue dots. (c) Red dots represent pixels within the central intensity *β* and its offset *ε*, whereas yellow and blue dots represent pixels with the intensity slightly lower and higher than red dots, respectively.

Select outer and inner rings for candidate points: Each candidate point in the multiresolution DoG pyramid is assigned with inner and outer rings as shown in Figure 6(a). We set the size of the rings according to [28] in which the inner ring consists of 8 pixels whereas the outer ring consists of 16 pixels circling the candidate point with a radius of 3 pixels. The candidate points *p*_*i*_(*x*_*i*_, *y*_*i*_) are pixels in the DoG image within the following spatial position:

(3) $$\begin{array}{c} x_{i}\in \left[4,M-3\right],\\ y_{i}\in \left[4,N-3\right], \end{array}$$

where  *x*_*i*_ is the spatial position of the candidate point at  *x*-axis, *y*_*i*_ is the spatial position of the candidate point at *y*-axis, *M* is the width of the image, and *N* is the height of the image.

**Step 3 .**

Test the inner ring patterns for candidate points: Patterns for the inner ring test consider four out of eight inner ring pixels wherein two pixels in one direction should be brighter than the other two pixels in the orthogonal direction. These patterns will be in the shape of × and +. All possible patterns for each shape are shown in Figure 6(b). The candidate point can pass the inner ring test with one or both shapes × and +. Then, the central intensity value *β* is estimated based on the median value of four pixels if the inner ring test is passed with one shape. Eight pixels will be considered for *β* if the inner ring test is passed with two shapes. The inner ring test will eliminate approximately 80% of the candidate points.

**Step 4 .**

Test the outer ring patterns for candidate points: The outer ring denoted by *B* = {*b*_*j*_ | *j* = 1,…, 16} is a circle with a circumference of 16 pixels and candidate point at the center. The intensity of *B* denoted by *I*_*b*_*j*__ in the outer ring can be divided into three labels *L*, namely, *d* (yellow dot), *s* (red dot), and *l* (blue dot) based on the central intensity value *β* and offset *ε* as follows:

(4) $$\begin{array}{c} L_{bj}=\left\{\begin{array}{c} d,\;I_{bj}<\beta -\varepsilon \\ s,\;\beta -\varepsilon \leq I_{bj}\leq \beta +\varepsilon \\ l,\;I_{bj}>\beta +\varepsilon . \end{array}\right. \end{array}$$

Label *s* represents pixels with the intensity within the neighborhood of central intensity value *β* and its offset *ε*. Label *d* and *l* represent pixels with the intensity slightly lower and higher than label *s*, respectively. In this study, the offset value *ε* is empirically set to 0.0010 as we used the absolute image with the pixel values in the range of [0, 1] where black has a value of zero and white as one. Based on these labels, the candidate point passes the test if its outer ring contains a consecutive and alternating arcs of label *d* and *l*. The length of these arcs should be in between 2 to 8 pixels [28]. An exception is given if the arcs of label *d* and *l* are separated by label *s* up to 2 pixels. Four examples of possible outer ring patterns are depicted in Figure 6(c). The details regarding the inner and outer ring tests can be found in [28].

**Step 5 .**

Apply nonmaxima suppression: Nonmaxima suppression with 3-by-3 neighborhood is applied to the candidate point that passes both inner and outer ring tests. However, the nonmaxima suppression is only applied to the candidate point within the first octave of the DoG pyramid. This is because the feature in higher octave is relatively coarse due to successive downsampling.

**Step 6 .**

Position refinements to estimate subpixel precision for each point that passes the tests [28].

**Step 7 .**

Finally, the spatial positions of the feature points detected in all octaves are estimated in the coordinate system of the input image.

#### 2.1.2. Stage 2–Stage 4: Descriptor, Matching, and Geometrical Transformation

In stage 2, the histogram of oriented gradients (HOG) descriptor [31] is computed for each D-Saddle point extracted in stage 1. The size of the cell in HOG is 8-by-8 pixels while the block is 2-by-2 cells. Then, in stage 3, the descriptors are matched between the image pair using approximate nearest neighbor search [32] with a ratio threshold of 0.9 to find the corresponding points.

After the matching process, the outliers are excluded from the corresponding points between the image pair using MSAC algorithm [15]. For MSAC, maximum number of random trials to find the inliers is set to 8000 and maximum distance between transformed points in the moving image to its corresponding points in the fixed image is set to four values: 1, 20, 60, and 80 pixels. The inliers obtained from this process are employed in estimating the transformation between the image pair. However, due to the randomized nature of MSAC algorithm, the transformation estimated differed between iterations. Therefore, the MSAC algorithm and transformation process are repeated 4000 times for each pair (1000 times for each maximum distance value) to ensure its convergence. From this repetition, the best registration accuracy in each pair is selected as the final result.

In stage 4, three models of transformation are utilized in the registration: similarity, affine, and second-order polynomial function. The transformation model is selected based on the number of inliers. In challenging image when the number of inliers is less than 8, similarity transformation is selected. Affine transformation is selected when the number of inliers is more than 8 and less than 30. If the number of inliers is more than 30, a second-order polynomial function is selected [33].

### 2.2. Dataset

Registration performance of D-Saddle is tested on Fundus Image Registration (FIRE) Dataset [34, 35], the only publicly available retinal image registration dataset with ground truth annotation. The retinal images were acquired using a Nidek AFC-210 fundus camera with resolution of 2912 × 2912 pixels and 45° field of view (FOV). The dataset consists of 134 retinal image pairs and classified into three categories according to their registration application, that is, super-resolution (category *𝒮*), image mosaicking (category *𝒫*), and longitudinal study (category *𝒜*). The details characteristics of each category in FIRE dataset are summarized in Table 1.

Category *𝒮* and category *𝒫* consist of image pairs with pathological cases but the anatomical appearance remains unchanged between image pairs. Category *𝒜* consists of image pairs with anatomical changes due to progression or remission of retinopathy. The changes include the variations of vessel tortuosity, microaneurysms, cotton-wool, and spots between image pairs. Category *𝒫* has the smallest overlap between image pairs that range between 17% and 89% and the largest range of rotation between 0° and 7° among the categories.

Three metrics are used to perceive image quality of FIRE dataset, namely, mean squared error (MSE), structural similarity index (SSIM) [36], and peak deviation nonuniformity (UN) [37]. MSE and SSIM measure the similarity between the image pair. MSE perceives the intensity difference whereas SSIM describes the similarity of the structure component. A higher similarity between the images is approximated by a lower MSE value and a value close to 1 for SSIM. UN measures the uniformity of intensity in an image where a higher UN value indicates a more uniform image. UN quantifies the intensity uniformity in an image based on maximum and minimum pixel values within the region of interest (ROI). This makes UN sensitive to nonuniformities such as illumination artifact near the frame boundary or dark spot artifact. We describe the intensity uniformity in the image pair through averaging UN values from two images.

**Figure 7:**
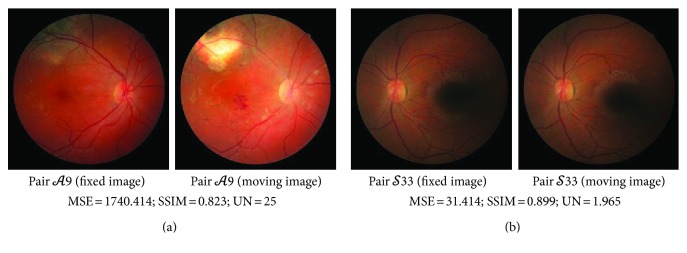
Examples of retinal image pairs with (a) high-intensity difference and (b) nonuniform intensity in FIRE dataset.

Retinal image pairs in category *𝒜* attained the biggest range of MSE and UN values representing variations of intensity difference and uniformity between image pairs in the category. These variations which are caused by certain images in category *𝒜* are acquired at different examination time under different lighting conditions. All categories have a high and comparable SSIM value within the range of 0.784 to 0.939 indicating a high similarity of structure component between image pairs and minimal blurring effect on vessel edges due to motion artifact or improper focusing. Examples of image pairs with high intensity difference and nonuniform intensity in the dataset are shown Figure 7().

### 2.3. Evaluation Criteria

There are two main aspects evaluated in this study. First, we assess the feature detection in Saddle and D-Saddle by comparing the feature points detected, matched, total inliers, and running time. Second, registration performance of the proposed D-Saddle is compared against four state-of-the-art retinal image registration methods GDB-ICP [25], Harris-PIIFD [20], H-M [24], and Saddle [28]. The experimental results for GDB-ICP, Harris-PIIFD, and H-M that tested on FIRE dataset are obtained from [34, 35]. We utilized the same registration accuracy measurement and ground truth as these methods. Furthermore, we only compare the registration performance with GDB-ICP, Harris-PIIFD, and H-M; thus, variation of registration performance due to different platform or hardware implementation in [34, 35] is minimal. Experiments for Saddle and D-Saddle are implemented in MATLAB running on Intel® Core™ i7-4770 CPU@3.40 GHz 8.00 GB RAM while the experimental results for GDB-ICP, Harris-PIIFD, and H-M are obtained from [34, 35]. The same offset value *ε*, descriptor, matching, and transformation described earlier are implemented for Saddle and D-Saddle. Additionally, the image pairs are processed in 583 × 583 resolution to reduce the cost of processing but evaluated according to the original resolution in the dataset to ensure a fair comparison with GDB-ICP, Harris-PIIFD, and H-M.

We evaluate the registration performance of the proposed D-Saddle and state-of-the-art methods on FIRE dataset using a set of evaluation metrics described as follows.

(1) Registration accuracy—we measure target registration error (TRE) to describe the registration accuracy of a method in which, a high registration accuracy is represented by a small TRE value and vice versa. TRE is an average distance measured in pixel from 10 corresponding landmarks (*n*) or ground truth between fixed (*x*_1_, *y*_1_) and moving (*x*_2_, *y*_2_) images after registration expressed in (5). The landmarks identified by experts are provided by FIRE dataset.
  (5) $$\begin{array}{c} \frac{1}{10}\overset{n=10}{\underset{n=1}{\sum }}\sqrt{{\left(x_{2}-x_{1}\right)}^{2}-{\left(y_{2}-y_{1}\right)}^{2}}. \end{array}$$
(2) Successful registration—we consider registration with TRE value below 1 pixel as a successful registration for super-resolution application. For image mosaicking and longitudinal study applications, we consider TRE value below 5 pixels as this range is acceptable for clinical purposes [38]. Registration with TRE larger than these values for the respective application is considered as failed.
(3) *N*_success_—total of image pairs with successful registration.
(4) Success rate (%)—ratio of total image pairs with successful registration to the total of image pairs in the dataset.

## 3. Results and Discussion

### 3.1. Feature Detection

Saddle detected an average of 13939 feature points in an image with a standard deviation of 4714 points whereas D-Saddle detected 5876 feature points with a standard deviation of 967 points as listed in Table 2. A smaller standard deviation of feature points detected in D-Saddle compared to Saddle indicates that D-Saddle is more consistent and stable in detecting feature points between images.

**Figure 8:**
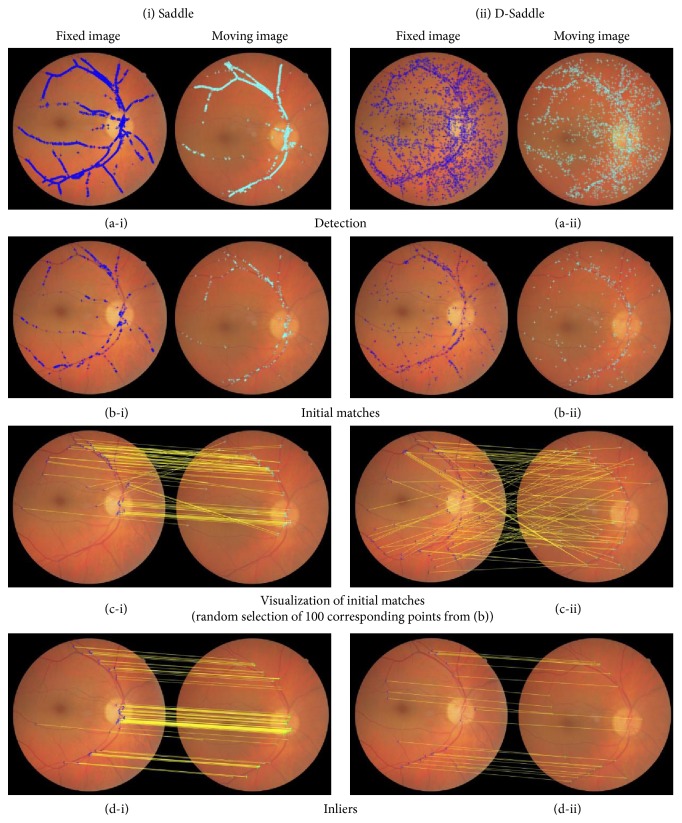
Example of Saddle and D-Saddle points (a) detected, (b) initial matched, (c) visualization of 100 randomly selected corresponding points, and (d) inliers on retinal image pair from category *𝒮*.

Saddle points are densely positioned on the strong contrast vessels (see Figure 8(a-i)) while D-Saddle points are distributed along the vessels of varying contrast and sizes (see Figure 8(a-ii)). The initial matches including the incorrect matches estimated by approximate nearest neighbor search [32] exclude 15% of Saddle points. The inliers after the removal of incorrect matches using MSAC algorithm constitute 6% of feature points detected by Saddle. In D-Saddle, the initial matches exclude 34% of the feature points detected and the inliers representing 8% of the feature points detected. Overall, the average of feature points detected, matched, and inliers in D-Saddle is approximately half from Saddle. However, a more accurate registration is estimated in D-Saddle as shown in the following subsection due to higher detection of D-Saddle points in low-quality region and distribution throughout the retinal image compared to Saddle.

Saddle took approximately 96 seconds to detect 13939 feature points while D-Saddle took 41 seconds to detect 5876 feature points in an image. D-Saddle requires half the time from Saddle as it detects 42% less feature points. Furthermore, D-Saddle is much faster to compute because the whole images in the multiresolution DoG pyramid represent 4/3 the size of the input image. In opposite, Saddle has to process six images with the same size as the input image to find the feature points.

### 3.2. Registration Performance

This section describes the registration performance of GDB-ICP, Harris-PIIFD, H-M, Saddle, and D-Saddle in FIRE dataset. We compute Spearman's rank-order correlation to measure the relationships between registration accuracy and factors such as percentage of overlapping area and image quality. For this analysis, TRE values from all image pairs are considered in the calculation. The Spearman's rank-order correlation is computed using IBM SPSS Statistics (version 24) software. The range of Spearman correlation is from −1 to +1 wherein the correlation is considered to be perfect when the correlation value is close to ±1 and weak when the correlation value is close to 0. The Spearman correlation is significant at the 0.05 level identified by a single asterisk or at the 0.01 level identified by two asterisks. Then, we compare the registration performance of D-Saddle with GDB-ICP, Harris-PIIFD, H-M, and Saddle according to the registration application in FIRE dataset.

#### 3.2.1. Percentage of Overlapping Area

The Spearman correlations between registration accuracy and percentage of overlapping area in FIRE dataset for all methods are summarized in Table 3. The registration accuracy of D-Saddle is negative and significantly correlated with the percentage of overlapping area (*r*_*s*_ = −0.795, *p* = <0.001). The registration accuracy of D-Saddle is rapidly decreased with the decreasing of overlapping area between the image pair. TRE of D-Saddle increases above 5 pixels when the overlapping area is below 75%. Furthermore, 83% of its failed registration is from image pairs with an overlapping area below 75%. Among all methods, Saddle has the strongest and significant correlation with the percentage of overlapping area (*r*_*s*_ = −0.819, *p* = <0.001). Saddle mainly failed to register image pairs when the overlapping area is below 87%. Another significant correlation between registration accuracy and percentage of overlapping area is observed in GDB-ICP with the weakest correlation among all methods (*r*_*s*_ = −0.588, *p* = <0.001).

#### 3.2.2. Image Quality

In fundus retinal imaging, image quality is highly dependent on the skills and experiences of the operator, settings of the camera, and cooperation from the patient. The common image quality degradations found in fundus retinal images are illumination artifact near the frame boundary, nonuniform intensity, image blurring due to motion artifact or improper focusing and dark spot artifact obscuring underlying tissues caused by poor dilated pupils. Measuring image quality of fundus retinal image is a subjective concept and varies between experts [39, 40]. Therefore, three metrics are used to measure retinal image quality in FIRE dataset, namely, MSE, SSIM, and UN to perceive the intensity difference between image pairs, similarity of structure component, and uniformity of intensity, respectively.

The Spearman correlation is computed to assess the influence of image quality on the registration accuracy of GDB-ICP, Harris-PIIFD, H-M, Saddle, and D-Saddle as summarized in Table 4. Harris-PIIFD, H-M, Saddle, and D-Saddle are positive and significantly correlated with MSE, which indicates that the registration accuracy decreases with the increase of intensity difference between the image pair. The registration accuracy of GDB-ICP shows no significant correlation with MSE. However, it should be noted that this correlation only computed from the registration with TRE values as some of the failed registrations in GDB-ICP yield no TRE values due to inability to estimate any transformations. The registration accuracy of H-M (*r*_*s*_ = −0.013, *p* = 0.878) has the weakest association with structure similarity between image pairs compared to other methods while D-Saddle (*r*_*s*_ = 0.001, *p* = 0.994) shown the weakest correlation among the methods with intensity uniformity.

In large overlapping retinal image pairs that exhibit illumination artifact near the frame boundary, significant nonuniform intensity, and dark spot artifact, D-Saddle successfully registered 43% of these image pairs with mean TRE of 0.857 pixel compared to GDB-ICP (17%), Harris-PIIFD (3%), H-M (20%), and Saddle (23%). This demonstrates that D-Saddle is more effective in registering low-quality images. Furthermore, a weaker correlation between registration accuracy and intensity uniformity is observed in D-Saddle compared to Saddle. D-Saddle suppressed nonuniform intensity through the DoG operator before searching for the feature points while Saddle considered the nonuniform intensity information within the ROI to search for the feature points.

#### 3.2.3. Overall Registration Performance

All registration accuracy of successful registrations for GDB-ICP, Harris-PIIFD, H-M, Saddle, and D-Saddle is summarized in Table 5. These results will demonstrate the ability of each method to register retinal images for various applications, that is, super-resolution (category *𝒮*), image mosaicking (category *𝒫*), and longitudinal study (category *𝒜*).

**Figure 9:**
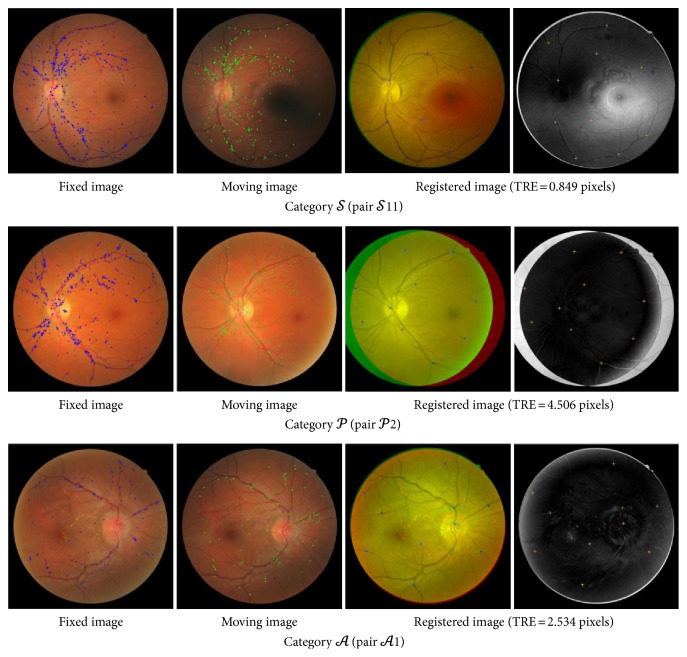
Examples of matched D-Saddle points between the image pair (1st and 2nd columns) and their registered image with landmarks (3rd and 4th columns) in each category of FIRE dataset. 3rd column: blended overlay image in which yellow represents areas with similar intensity. 4th column: difference in the image in which black represents areas with similar intensity.

In FIRE dataset, D-Saddle successfully registered 43% of retinal image pairs followed by GBD-ICP (28%), Saddle (16%), and H-M (16%). A lower overall success rate can be observed in Harris-PIIFD that failed to register a total of 129 retinal image pairs and contributed to the lowest overall success rate among all methods with only 4%. Examples of matched D-Saddle points between image pairs and their registered image for each category are depicted in Figure 9.

D-Saddle attained the highest success rate (86%) in longitudinal study application compared to its success rate in other categories. The mean TRE of successful registration is 3.896 ± 0.934 pixels with minimum TRE = 2.534 pixels from image pair *𝒜*1 and maximum TRE = 4.906 pixels from image pair *𝒜*6. D-Saddle failed to register image pair *𝒜*2 and *𝒜*8 that exhibit significant changes of vessel tortuosity and thickness between fixed and moving images. In these pairs, D-Saddle is unable to find the matches on the affected vessels as the corresponding points on these vessels are characterized by different descriptors. This is because HOG descriptor in D-Saddle pipeline characterizes the points with the surrounding structural information and can be sensitive in the presence of structural variation between the image pair. Consequently, the matches are mainly located and gathered on the unaffected vessels. Lack of matched distribution throughout the vessels has led to failed registration in these image pairs. The successful registration of GBD-ICP (29%), Harris-PIIFD (21%), H-M (29%), and Saddle (36%) in category *𝒜* is only seen in image pairs with minimal anatomical variations and mainly failed to register image pairs with variation of vessel tortuosity.

In super-resolution application, D-Saddle successfully registered 45% of the image pairs with mean TRE of 0.852 ± 0.116 pixels where its minimum TRE= 0.596 pixels is from image pair *𝒮*25 and maximum TRE = 0.999 pixels from image pair *𝒮*39. The mean TRE of successful registration of GBD-ICP, Harris-PIIFD, and H-M outperformed D-Saddle by 0.069, 0.006, and 0.013 pixel, respectively. However, a lower success rate is observed in GBD-ICP (24%), Harris-PIIFD (3%), and H-M (25%). Furthermore, GBD-ICP, Harris-PIIFD, H-M, and Saddle have shown to be more sensitive in registering retinal image pairs with the presence of illumination artifact near the frame boundary, significant nonuniform intensity, and dark spot artifact.

D-Saddle attained the lowest success rate (27%) in image mosaicking application of category *𝒫* with mean TRE of 4.520 ± 0.412 pixels compared to its success rate in other categories. This category contains a smaller overlap area compared to category *𝒮* and category *𝒜*. The minimum TRE of D-Saddle in this category is obtained from image pair *𝒫*19 with TRE of 3.741 pixels while the maximum TRE is obtained from image pair *𝒫*16 with TRE of 4.993 pixels. D-Saddle failed to register image pairs that exhibits surface wrinkling in which D-Saddle detects the feature points on both vessels and wrinkles that present the line-like structure. Consequently, the transformation estimated based on these points leads to failed registration. In this category, GBD-ICP recorded the highest success rate (33%) and the smallest mean TRE of successful registration (TRE = 3.068 ± 0.840) among all methods. Harris-PIIFD and H-M failed to register any of the image pairs in this category while Saddle successfully registered only 6% of the image pairs.

Then, we perform paired *t*-test to examine if registration accuracy in D-Saddle is significantly higher than Saddle in FIRE dataset. In this test, we consider TRE values of all image pairs in the dataset.  Table 6 shows that D-Saddle significantly outperforms Saddle in all categories. A bigger mean difference represents a greater improvement in D-Saddle registration accuracy. The mean difference is negative because D-Saddle has a smaller TRE compared to Saddle. The paired *t*-test shows that D-Saddle greatly improved Saddle performance in handling retinal image pairs with smaller overlap for image mosaicking application. The registration accuracy in D-Saddle is slightly higher than Saddle in the presence of anatomical difference between the image pairs in category *𝒜*. The smallest mean difference is observed in category *𝒮* which consists of retinal image pairs with large spatial overlap and similar anatomical appearances.

## 4. Conclusion

This paper introduces D-Saddle algorithm for the feature-based retinal image registration. D-Saddle incorporates the multiresolution DoG pyramid with Saddle detection module to improve its ability in detecting feature points on the low-quality region that consists of high and low contrast vessels of varying sizes. This is crucial to detect more distributed feature points on the retinal vessels and enable D-Saddle to register retinal image pairs for various registration applications such as super-resolution, image mosaicking, and longitudinal study applications.

D-Saddle is tested on FIRE dataset that consists of 134 retinal image pairs and categorized according to super-resolution, image mosaicking, and longitudinal study applications. We performed a comparative experiment between D-Saddle and four state-of-the-art retinal image registration methods GDB-ICP, Harris-PIIFD, H-M, and Saddle. Experimental results show that GDB-ICP attained higher registration accuracy than D-Saddle in all categories. However, D-Saddle successfully registered 43% of the retinal image pairs in FIRE dataset while a lower success rate can be observed in GDB-ICP (28%), Harris-PIIFD (4%), H-M (16%), and Saddle (16%). Furthermore, D-Saddle is more robust in registering retinal image pair compared to other methods for longitudinal study and super-resolution applications when it successfully registered 86% and 45% of the image pairs, respectively. In image mosaicking application, GDB-ICP successfully registered 33% of the image pairs and outperformed D-Saddle that successfully registered only 27% of the image pairs.

The registration accuracy of D-Saddle was shown to be influenced by the percentage of overlapping area between the image pair. Optimum performance of D-Saddle can be achieved when the overlap area is larger than 75%. This explained a lower success rate of D-Saddle in image mosaicking application compared to its success rate in other categories as 82% of the image pairs in this category has an overlap area smaller than 75%. However, D-Saddle is more effective in registering low-quality image pairs including illumination artifact near the frame boundary, significant nonuniform intensity, and dark spot artifact. In low-quality retinal image pairs with large overlapping area, D-Saddle successfully registered 43% of the image pairs whereas GDB-ICP, Harris-PIIFD, H-M, and Saddle only registered 17%, 3%, 20%, and 23% of the image pairs.

The paired *t*-test conducted between registration accuracy in D-Saddle and Saddle shows that D-Saddle significantly improved the registration accuracy of the original Saddle in all categories. The biggest improvement is observed in image mosaicking application while the smallest improvement is observed in the super-resolution application. In the future, we will concentrate on improving D-Saddle in retinal image pairs with smaller overlapping area and extend its implementation in other modalities.

## Figures and Tables

**Table 1:** Details of the characteristics of each category in FIRE dataset.

Details	Category *𝒮*	Category *𝒫*	Category *𝒜*
Total pairs	71	49	14
Application	Super-resolution	Mosaicking	Longitudinal study
Anatomical changes	No	No	Yes
Pathological cases	Yes	Yes	Yes
Resolution	2912 × 2912		
Scale factor^∗^	≈1	≈1	≈1
Overlap (%)	86–100	17–89	95–100
Rotation (°)	0°–5°	0°–7°	0°–4°
MSE	13–1541	109–1048	42–1728
SSIM	0.788–0.939	0.784–0.938	0.799–0.933
UN	7–771	55–524	21–864

^∗^Average scale factor (camera zoom or magnification) between retinal fundus image pairs.

**Table 2:** Details of feature points detected, matched, and inliers for Saddle and D-Saddle in FIRE dataset.

	Saddle			D-Saddle		
	Detect	Match	Inliers	Detect	Match	Inliers
Mean	13939	11850	904	5876	3906	447
Std. deviation	4714	4593	567	967	926	260
Min	4450	3234	73	3982	2071	64
Max	27047	24446	2443	8542	6501	1064
Running time (detect)	96 seconds			41 seconds		

**Table 3:** Correlation between registration accuracy and percentage of overlapping area in FIRE dataset.

		Registration accuracy				
		GDB-ICP	Harris-PIIFD	H-M	Saddle	D-Saddle
Overlapping area	Spearman's rho (*r_s_*)	−0.588	−0.763	−0.761	−0.819	−0.795
	*p* value (*p*)	<0.001^∗∗^	<0.001^∗∗^	<0.001^∗∗^	<0.001^∗∗^	<0.001^∗∗^

Correlation is significant at the 0.05 level. ^∗∗^Correlation is significant at the 0.01 level.

**Table 4:** Correlation between registration accuracy and image quality metrics in FIRE dataset.

		Registration accuracy				
		GDB-ICP	Harris-PIIFD	H-M	Saddle	D-Saddle
Intensity difference (MSE)	Spearman's rho (*r_s_*)	0.205	0.395	0.326	0.341	0.269
	*p* value (*p*)	0.064	<0.001^∗∗^	<0.001^∗∗^	<0.001^∗∗^	0.002^∗∗^
Structure similarity (SSIM)	Spearman's rho (*r_s_*)	−0.030	−0.094	−0.013	−0.071	−0.015
	*p* value (*p*)	0.788	0.288	0.878	0.417	0.863
Intensity uniformity (UN)	Spearman's rho (*r_s_*)	0.193	−0.093	0.037	0.016	0.001
	*p* value (*p*)	0.083	0.297	0.673	0.853	0.994

Correlation is significant at the 0.05 level. ^∗∗^Correlation is significant at the 0.01 level.

**Table 5:** Details of success rate and registration accuracy of successful registration for GDB-ICP, Harris-PIIFD, H-M, Saddle, and D-Saddle in FIRE dataset.

Methods	*N*_success_	Success rate (%)	Registration accuracy (TRE) of successful registration (pixels)					^∗^Std. deviation of all pairs
			Mean	95% confidence interval of the mean		Min	Max	
				Lower	Upper			
Overall								
GDB-ICP	37	28%	1.988	1.566	2.411	0.486	4.952	1.479
Harris-PIIFD	5	4%	2.573	0.571	4.576	0.785	4.244	365.754
H-M	22	16%	1.232	0.849	1.616	0.554	3.315	39.918
Saddle	21	16%	2.059	1.299	2.818	0.719	4.977	7.553
D-Saddle	57	43%	2.329	1.862	2.797	0.596	4.993	3.988
Category *𝒮* (super-resolution)								
GDB-ICP	17	24%	0.783	0.703	0.863	0.486	0.988	0.778
Harris-PIIFD	2	3%	0.846	0.071	1.621	0.785	0.907	1.969
H-M	18	25%	0.839	0.780	0.897	0.554	0.995	1.886
Saddle	13	18%	0.858	0.806	0.911	0.719	0.963	1.265
D-Saddle	32	45%	0.852	0.810	0.894	0.596	0.999	0.897
Category *𝒫* (image mosaicking)								
GDB-ICP	16	33%	3.068	2.620	3.516	1.946	4.952	1.134
Harris-PIIFD	0	0%	N/A	N/A	N/A	N/A	N/A	580.486
H-M	0	0%	N/A	N/A	N/A	N/A	N/A	18.980
Saddle	3	6%	4.921	4.785	5.058	4.867	4.977	4.532
D-Saddle	13	27%	4.520	4.271	4.769	3.741	4.993	3.301
Category *𝒜* (longitudinal study)								
GDB-ICP	4	29%	2.792	1.903	3.680	2.354	3.603	3.444
Harris-PIIFD	3	21%	3.725	2.551	4.899	3.319	4.244	396.753
H-M	4	29%	3.004	2.664	3.343	2.857	3.315	110.853
Saddle	5	36%	3.462	2.495	4.429	2.767	4.769	4.277
D-Saddle	12	86%	3.896	3.303	4.490	2.534	4.906	3.637

^∗^Standard deviation of TRE from all image pair (both failed and successful registration).

**Table 6:** Paired *t*-test between registration accuracy of D-Saddle and Saddle.

Paired differences D-Saddle−Saddle							
	Mean difference	Std. deviation	95% confidence interval of the difference		*t*	df	*p* value
			Lower	Upper			
Overall	−3.360	4.252	−4.087	−2.634	−9.147	133	<0.001^∗^
Category *𝒮*	−0.371	0.434	−0.473	−0.268	−7.192	70	<0.001^∗^
Category *𝒫*	−8.214	3.314	−9.166	−7.262	−17.352	48	<0.001^∗^
Category *𝒜*	−1.532	1.485	−2.389	−0.675	−3.861	13	0.002^∗^

^∗^Significant at the 0.05 level.

## References

[1] Brown D. M., Ciardella A. (2005). Mosaic fundus imaging in the diagnosis of retinal diseases. *Investigative Ophthalmology & Visual Science*.

[2] Bontala A., Sivaswamy J., Pappuru R. R. Image mosaicing of low quality neonatal retinal images.

[3] Lee B. H., Xu G., Gopalakrishnan K. AEGIS-augmented eye laser treatment with region guidance for intelligent surgery.

[4] Adal K. M., van Etten P. G., Martinez J. P., van Vliet L. J., Vermeer K. A. (2015). Accuracy assessment of intra- and intervisit fundus image registration for diabetic retinopathy screening accuracy assessment of fundus image registration. *Investigative Ophthalmology & Visual Science*.

[5] Legg P. A., Rosin P. L., Marshall D., Morgan J. E. (2013). Improving accuracy and efficiency of mutual information for multi-modal retinal image registration using adaptive probability density estimation. *Computerized Medical Imaging and Graphics*.

[6] Kolar R., Sikula V., Base M. (2010). Retinal image registration using phase correlation. *Analysis of Biomedical Signals and Images*.

[7] Reel P. S., Dooley L. S., Wong K. C. P., Börner A. Enhanced retinal image registration accuracy using expectation maximisation and variable bin-sized mutual information.

[8] Liu C. Y., Ma J. Y., Ma Y., Huang J. (2016). Retinal image registration via feature-guided Gaussian mixture model. *Journal of the Optical Society of America A*.

[9] Chen L., Huang X. T., Tian J. (2015). Retinal image registration using topological vascular tree segmentation and bifurcation structures. *Biomedical Signal Processing and Control*.

[10] Chen L., Xiang Y., Chen Y. J., Zhang X. L. Retinal image registration using bifurcation structures.

[11] Perez-Rovira A., Cabido R., Trucco E., McKenna S. J., Hubschman J. P. (2012). RERBEE: robust efficient registration via bifurcations and elongated elements applied to retinal fluorescein angiogram sequences. *IEEE Transactions on Medical Imaging*.

[12] Shen B., Zhang D. B., Peng Y. H. (2012). Blood bifurcation structure and global to local strategy based retinal image registration. *Pattern Recognition*.

[13] Patankar S. S., Kulkarni J. V. (2015). Orthogonal moments for determining correspondence between vessel bifurcations for retinal image registration. *Computer Methods and Programs in Biomedicine*.

[14] Fischler M. A., Bolles R. C. (1981). Random sample consensus: a paradigm for model fitting with applications to image analysis and automated cartography. *Communications of the ACM*.

[15] Torr P. H., Zisserman A. (2000). MLESAC: a new robust estimator with application to estimating image geometry. *Computer Vision and Image Understanding*.

[16] Harris C., Stephens M. A combined corner and edge detector.

[17] Bay H., Tuytelaars T., Van Gool L. SURF: speeded up robust features.

[18] Bay H., Ess A., Tuytelaars T., Van Gool L. (2008). Speeded-up robust features (SURF). *Computer Vision and Image Understanding*.

[19] Lowe D. G. (2004). Distinctive image features from scale-invariant keypoints. *International Journal of Computer Vision*.

[20] Chen J., Tian J., Lee N., Zheng J., Smith R. T., Laine A. F. (2010). A partial intensity invariant feature descriptor for multimodal retinal image registration. *IEEE Transactions on Biomedical Engineering*.

[21] Wang G., Wang Z. C., Chen Y. F., Zhao W. D. (2015). Robust point matching method for multimodal retinal image registration. *Biomedical Signal Processing and Control*.

[22] Saha S. K., Xiao D., Frost S., Kanagasingam Y. (2016). A two-step approach for longitudinal registration of retinal images. *Journal of Medical Systems*.

[23] Hernandez-Matas C., Zabulis X., Argyros A. A. Retinal image registration based on keypoint correspondences, spherical eye modeling and camera pose estimation.

[24] Hernandez-Matas C., Zabulis X., Argyros A. A. Retinal image registration through simultaneous camera pose and eye shape estimation.

[25] Yang G., Stewart C. V., Sofka M., Tsai C.-L. (2007). Registration of challenging image pairs: initialization, estimation, and decision. *IEEE Transactions on Pattern Analysis and Machine Intelligence*.

[26] Ghassabi Z., Shanbehzadeh J., Sedaghat A., Fatemizadeh E. (2013). An efficient approach for robust multimodal retinal image registration based on UR-SIFT features and PIIFD descriptors. *Eurasip Journal on Image and Video Processing*.

[27] Ghassabi Z., Shanbehzadeh J., Mohammadzadeh A., Ostadzadeh S. S. (2015). Colour retinal fundus image registration by selecting stable extremum points in the scale-invariant feature transform detector. *IET Image Processing*.

[28] Aldana-Iuit J., Mishkin D., Chum O., Matas J. In the saddle: chasing fast and repeatable features.

[29] Marr D. (1982). *Vision*.

[30] Marr D., Hildreth E. (1980). Theory of edge detection. *Proceedings of the Royal Society of London B: Biological Sciences*.

[31] Dalal N., Triggs B. Histograms of oriented gradients for human detection.

[32] Muja M., Lowe D. G. Fast approximate nearest neighbors with automatic algorithm configuration.

[33] Ghassabi Z., Shanbehzadeh J., Mohammadzadeh A. (2016). A structure-based region detector for high-resolution retinal fundus image registration. *Biomedical Signal Processing and Control*.

[34] Hernandez-Matas C., Zabulis X., Triantafyllou A., Anyfanti P., Douma S., Argyros A. A. (2016). *FIRE: Fundus Image Registration Dataset*.

[35] Hernandez-Matas C., Zabulis X., Triantafyllou A., Anyfanti P., Douma S., Argyros A. A. (2017). FIRE: fundus image registration dataset. *Journal for Modeling in Ophthalmology*.

[36] Wang Z., Bovik A. C., Sheikh H. R., Simoncelli E. P. (2004). Image quality assessment: from error visibility to structural similarity. *IEEE Transactions on Image Processing*.

[37] Goerner F. L., Duong T., Stafford R. J., Clarke G. D. (2013). A comparison of five standard methods for evaluating image intensity uniformity in partially parallel imaging MRI. *Medical Physics*.

[38] Matsopoulos G. K., Asvestas P. A., Mouravliansky N. A., Delibasis K. K. (2004). Multimodal registration of retinal images using self organizing maps. *IEEE Transactions on Medical Imaging*.

[39] Giancardo L., Abràmoff M. D., Chaum E., Karnowski T., Meriaudeau F., Tobin K. Elliptical local vessel density: a fast and robust quality metric for retinal images.

[40] Bartling H., Wanger P., Martin L. (2009). Automated quality evaluation of digital fundus photographs. *Acta Ophthalmologica*.

